# Supplementary material for: Influence of PM2.5 Exposure Level on the Association between Alzheimer’s Disease and Allergic Rhinitis: A National Population-Based Cohort Study
Source: Int J Environ Res Public Health. 2019 Sep 11;16(18):3357. doi: 10.3390/ijerph16183357 (PMC6765937; doi:10.3390/ijerph16183357)
Supplement: Supplementary file 1 [file ijerph-16-03357-s001.pdf]

Table S1. Probit regression analysis of $PM_{2.5}$ and AR in patients with AD.

Variable	AD		
	**Estimated Coefficient (95% CI)**	**Std. Error**	***p***
PM2.5 exposure level (reference: Q1 level)			
Q2 level	0.327 (0.096–0.557)	0.118	0.0050
Q3 level	0.474 (0.249–0.699)	0.115	<0.0001
Q4 level	0.622 (0.402–0.843)	0.113	<0.0001

Adjusted gender, age, low income, urbanization level, comorbidity; Abbreviation: Std. Error—Standard error.
